# Hepcidin as a Biomarker of Response to Antifibrotic Therapy in Idiopathic Pulmonary Fibrosis

**DOI:** 10.3390/jcm15187023

**Published:** 2026-09-10

**Authors:** Gulcin Yilmaz Gunes, Hikmet Çoban, Fuat Erel, Merve Akış Yılmaz, Nurhan Sarioglu, Mustafa Colak, Merve Yumrukuz Senel

**Affiliations:** 1 Department of Pulmonology, Bandırma Training and Research Hospital, Balıkesir 10200, Türkiye; 2 Department of Pulmonology, Faculty of Medicine, Balıkesir University, Balıkesir 10145, Türkiye; hikmet.coban@balikesir.edu.tr (H.Ç.);; 3 Department of Medical Biochemistry, Faculty of Medicine, Balıkesir University, Balıkesir 10145, Türkiye

**Keywords:** idiopathic pulmonary fibrosis, hepcidin, antifibrotic therapy, biomarker

## Abstract

**Background/Objectives:** Idiopathic pulmonary fibrosis (IPF) may progress despite antifibrotic therapy, highlighting the need for biomarkers of early treatment-associated biological changes. This study aimed to assess the potential role of hepcidin in monitoring treatment response by evaluating pre- and post-treatment serum hepcidin levels in patients with IPF receiving antifibrotic therapy. **Methods:** This prospective observational cohort study included 38 clinically stable patients diagnosed with IPF according to the 2022 American Thoracic Society/European Respiratory Society (ATS/ERS)criteria between January and July 2025. Serum hepcidin was measured by ELISA at antifibrotic therapy initiation and month 3. Demographic data, pulmonary function tests, diffusing capacity for carbon monoxide (DLCO), and 6 min walk test results were recorded. Analyses were performed using SPSS 25.0. **Results:** Mean serum hepcidin decreased from 33.00 ± 16.74 to 24.41 ± 12.42 ng/mL (*p* < 0.001), with reductions observed in 30 of 38 patients (78.9%). Mean forced vital capacity (FVC) increased from 73.83 ± 18.10% predicted to 77.74 ± 15.70% predicted (*p* < 0.001). Median DLCO increased from 68.50 (51.00–80.50)% predicted to 72.50 (60.00–83.75)% predicted (*p* < 0.001). Six-minute walk distance did not change significantly (*p* = 0.078), nor did hemoglobin, C-reactive protein, or erythrocyte sedimentation rate. The change in hepcidin did not differ between the pirfenidone and nintedanib groups (*p* = 0.817). Although FVC and DLCO increased over three months, these short-term functional changes should be interpreted cautiously, as test familiarization, measurement variability, and regression to the mean cannot be excluded; therefore, they should not be considered evidence of fibrosis reversal or a direct treatment effect. **Conclusions:** Our study provides the first real-world data demonstrating a significant decrease in serum hepcidin following antifibrotic treatment in patients with IPF. These findings suggest that serum hepcidin may have potential clinical utility as a biomarker for assessing response to antifibrotic therapy in IPF.

## 1. Introduction

Idiopathic pulmonary fibrosis (IPF) is a chronic interstitial lung disease with a poor prognosis, characterized by progressive fibrosis (scarring) in the lung parenchyma. In patients diagnosed with IPF, median survival is limited to approximately 3–5 years, and annual mortality and morbidity rates increase markedly as the disease progresses [1,2]. In the untreated period, the rate of decline in lung function (FVC) is around 10% per year on average [3]. The clinical course is heterogeneous, and episodes of sudden deterioration such as acute exacerbations contribute significantly to morbidity and mortality [4]. Therefore, antifibrotic therapies aimed at slowing the progression of the disease are of critical importance.

Pirfenidone and nintedanib are approved antifibrotic drugs in the treatment of IPF and have been in clinical use since 2014 [5,6]. In randomized clinical trials, these drugs reduced the rate of FVC decline by approximately half compared with placebo over one year of treatment and significantly reduced the risk of acute exacerbation [5,6].

However, these treatments have not been able to completely stop fibrosis and have only been able to slow the rate of disease progression [5,6]. Nevertheless, IPF may progress despite current antifibrotic therapies, and in clinical practice, treatment response and the rate of progression may differ markedly among individuals [7]. Because of this limited effect, there is a need for sensitive biomarkers that can monitor the “silent” progression of IPF and predict response to treatment [8,9].

Although current candidate biomarkers include molecules such as surfactant protein D (SP-D), Krebs von den Lungen-6 (KL-6), and matrix metalloproteinase-7 (MMP-7), their reliability and clinical use in disease follow-up are still limited [8,10]. Levels of these biomarkers are generally found to be high in patients with IPF and have been associated with disease severity or prognosis; however, none is sufficiently specific or reliable to enter routine practice [11]. Therefore, there remains a need for new and more sensitive biomarkers directly related to IPF pathophysiology. In this context, targets such as fibroblast activation protein (FAP) have been emphasized in recent years; it has been shown that this protein, expressed on the surface of active fibroblasts, may reflect fibrotic activity both through circulating levels and through specific imaging methods [11]. However, there is still no specific biomarker that has entered clinical use to reliably monitor IPF progression.

Because systemic inflammation and immune dysregulation contribute to the clinical course in idiopathic pulmonary fibrosis (IPF) and other interstitial lung diseases, composite inflammation indices derived from routine laboratory tests have been evaluated with increasing frequency in recent years. In this context, plateletcrit (PCT), systemic immune-inflammation index (SII), the hemoglobin-albumin-lymphocyte-platelet (HALP) score, and the index presented as PIV in our study but more commonly referred to in the literature as the pan-immune-inflammation value (PIV) are low-cost and easy-to-apply indicators that can be calculated from complete blood count and basic biochemistry parameters. The use of these indices in the context of IPF/ILD is also increasing; for example, it has been reported that SII may provide discriminative/clinical information in IPF and different ILD subgroups and may be associated with mortality in IPF [12,13]. Similarly, there are studies reporting that the HALP score may be associated with mortality/prognosis in IPF [14,15].

A recent study showed that serum hepcidin levels in patients with IPF were significantly higher than those in healthy controls. In the IPF group, hepcidin level was found not to be associated with the presence of anemia or systemic inflammation indicators. These data suggest that the increase in hepcidin may be disease-specific and suggest that hepcidin may be a new biomarker candidate in patients with IPF [16].

However, there are insufficient data in the clinical literature regarding the change in serum hepcidin levels during the treatment process in patients with IPF receiving antifibrotic therapy (pirfenidone or nintedanib). Accordingly, the main hypothesis of our study is that a significant decrease in serum hepcidin levels will occur due to suppression of inflammation and fibrotic activity with antifibrotic therapy. To test this hypothesis, serum hepcidin levels of patients diagnosed with IPF who were followed prospectively were compared before and after antifibrotic therapy.

## 2. Materials and Methods

### 2.1. Study Design

This single-center prospective observational cohort study was conducted in the Chest Diseases Clinic of Balıkesir University Health Practice and Research Hospital between January 2025 and July 2025. Ethics Committee approval was obtained before study initiation, on 17 December 2024 (decision no. 2024/236), and participant recruitment and all study-related procedures commenced only after this approval. The decision to initiate antifibrotic therapy and the choice of pirfenidone or nintedanib were made by the treating physician as part of routine clinical care and were not determined by the study protocol. Participants were not prospectively assigned by the investigators to an intervention or comparator group.

Thirty-eight patients with IPF who were diagnosed according to the American Thoracic Society/European Respiratory Society (ATS/ERS)2022 criteria and were in the stable period were included in the study. The inclusion criteria for patients with idiopathic pulmonary fibrosis (IPF) were patients who were diagnosed with IPF according to the ATS/ERS 2022 guidelines and in the stable phase of the disease. The exclusion criteria were an acute exacerbation of IPF within the preceding three months, the presence of an infectious or rheumatological disease, hepatic or renal failure, and any known, pathologically confirmed malignancy. The Charlson Comorbidity Index (CCI) was calculated to evaluate comorbidities for the IPF group. Nutritional status and gastrointestinal adverse effects were clinically monitored throughout the follow-up period. Patients who developed major gastrointestinal adverse effects requiring a change in antifibrotic therapy were excluded from the final analysis.

Demographic data (age, smoking habits, and comorbid diseases) of the IPF patients included in the study were recorded.

Pulmonary function assessments were performed at baseline and at month 3 in the same pulmonary function laboratory, using the same equipment at both visits, while patients were clinically stable. Flow–volume spirometry was performed using an nSpire/ZAN pulmonary function testing system (nSpire Health GmbH, Oberthulba, Germany). Diffusing capacity for carbon monoxide was measured in the seated position using the single-breath technique with a Quark PFT system equipped with a 28-mm turbine flowmeter (COSMED S.r.l., Albano Laziale, Italy). FVC and DLCO results were expressed as percentages of predicted values, and Global Lung Function Initiative reference equations were used for DLCO. The 6 min walk distance was assessed at both visits and recorded in meters.

Patients with IPF were divided into two groups according to anemia status. Anemia was defined as hemoglobin levels < 12 g/dL in women and <13 g/dL in men. To evaluate the etiology of anemia, serum ferritin, iron, unsaturated iron-binding capacity (UIBC), transferrin saturation, vitamin B12, folate, and erythropoietin (EPO) levels were measured.

All procedures of this study were carried out in accordance with the Declaration of Helsinki, and approval was obtained from the Balıkesir University Rectorate Health Sciences Ethics Committee (Ethics Committee date: 17 December 2024, decision no: 2024/236). Written informed consent was obtained from all participants included in the study.

### 2.2. Laboratory Assessments

Blood samples were obtained from all participants in the IPF group before treatment and in the third month after initiation of treatment, after an overnight fast, for complete blood count and serum analysis. Given the high mortality associated with IPF and the importance of identifying early treatment-associated changes, the three-month follow-up time point was selected to evaluate early changes in serum hepcidin levels following the initiation of antifibrotic therapy. This interval was not intended to assess structural regression or long-term progression of pulmonary fibrosis; longer follow-up is required to determine the durability and long-term clinical relevance of the observed changes. After waiting for the blood collected in yellow-top gel tubes to clot, serum was separated and then centrifuged at +4 °C for 10 min at 4000 rpm. A portion of the serum was aliquoted into Eppendorf tubes and stored at −40 °C for hepcidin analysis.

Complete blood count was performed in the collected blood samples using a DxH 800 hematology analyzer (Beckman Coulter, Brea, CA, USA). Serum iron and unsaturated iron-binding capacity (UIBC) levels were measured using an AU680 analyzer (Beckman Coulter, Brea, CA, USA). Serum ferritin, vitamin B12, and folate levels were evaluated using a UniCel DxI 600 analyzer (Beckman Coulter, Brea, CA, USA).

Serum C-reactive protein (CRP) levels were measured using a BN II analyzer (Siemens Healthineers, Marburg, Germany), and erythrocyte sedimentation rate (ESR) was measured using an ALS-100 analyzer (Alaris, İzmir, Turkey).

Total iron-binding capacity (TIBC) was calculated using the formula [TIBC (μg/dL) = serum iron (μg/dL) + UIBC (μg/dL)]. Transferrin saturation percentage was calculated using the formula [TSAT (%) = serum iron (μg/dL)/TIBC (μg/dL) × 100]. As defined in the literature, PCT = platelet count × mean platelet volume (MPV)/10,000, SII = neutrophil × platelet/lymphocyte, PIV = neutrophil × platelet × monocyte/lymphocyte, and HALP = hemoglobin × albumin × lymphocyte/platelet were calculated using the formulas [17,18,19,20]. All analyses were performed in the Biochemistry and Microbiology Laboratory of Balıkesir University Health Practice and Research Hospital.

Serum EPO levels were analyzed at Gelişim Medical Laboratory using the Siemens Immulite 2000 XPi immunoassay system (Siemens Healthcare Diagnostics Inc., Flanders, NJ, USA).

### 2.3. Serum Hepcidin Analysis

Serum hepcidin levels were determined using a commercially available enzyme-linked immunosorbent assay (ELISA) kit (Human Hepcidin ELISA, Elabscience, Catalog No: E-EL-H6202, Houston, TX, USA).

Hepcidin measurement was performed spectrophotometrically at a wavelength of 450 nm using a multimode plate reader (Varioskan Flash Multimode Reader, Thermo Scientific, Waltham, MA, USA). The measurement range of the kit is 0.78–50 ng/mL, the sensitivity is 0.32 ng/mL, and the coefficient of variation is less than 10%. All procedures were performed in accordance with the manufacturer’s instructions. Serum hepcidin measurement was performed in the Medical Biochemistry Laboratory of Balıkesir University Faculty of Medicine. Samples yielding results above the measurement range were diluted at appropriate ratios in accordance with the manufacturer’s instructions and re-assayed, and the final concentration was corrected by the dilution factor.

### 2.4. Statistical Analysis

For statistical evaluation of the data obtained in the study, the SPSS 25.0 software package (SPSS Inc., Chicago, IL, USA) was used. Descriptive statistics for continuous variables were expressed as mean ± standard deviation or median (minimum–maximum range), according to their distribution. Categorical variables were expressed as number and percentage (%).

Normality of the data was evaluated using the Shapiro–Wilk test. For data with a normal distribution, comparisons between pre- and post-treatment measurements were performed using the paired-samples *t*-test. Differences between pre- and post-treatment values of data that were not normally distributed were analyzed using the Wilcoxon signed-rank test. For comparison of anemic and non-anemic groups, the independent-samples *t*-test (Welch *t*-test when homogeneity of variances was not met) was used for normally distributed variables, and the Mann–Whitney U test was used for non-normally distributed variables. Associations between two continuous variables were evaluated using Spearman correlation analysis.

The primary endpoint, the change in serum hepcidin level, was evaluated using the paired-samples *t*-test by pairing pre- and post-treatment measurements (two-sided).

In statistical evaluations, the significance level was accepted as *p* < 0.05, and two-sided *p* values were used in all comparisons. Results are presented together with relevant descriptive statistics and *p* values. An a priori sample size calculation was performed using G*Power version 3.1.9.4 for a two-sided paired-samples *t*-test. Assuming an anticipated standardized paired effect size of Cohen’s dz = 0.60, an alpha level of 0.05, and 95% statistical power, the required sample size was 39 participants (theoretical power: 95.46%). The final analyzable cohort included 38 participants, corresponding to a theoretical power of 94.96% under the same assumptions. Because a standardized paired effect size was used, raw baseline variance was not entered separately; variability was incorporated through the standard deviation of the within-participant differences.

## 3. Results

Table 1 shows that a total of 38 patients with idiopathic pulmonary fibrosis (IPF) were included in the study. The mean age of the patients was 71.47 ± 6.94 years, and 20 (52.6%) were male and 18 (47.4%) were female. Anemia was detected in 8 patients (21.1%) according to WHO criteria. In patients with a smoking history, the pack-year value was calculated as the median of 15.00 (0.00–46.25). The median Charlson Comorbidity Index was 3.00 (3.00–4.00). In pulmonary function tests at treatment initiation, the mean FVC value was 73.83 ± 18.10%, the mean FEV1 value was 82.22 ± 19.68%, and the FEV1/FVC ratio was 85.61 ± 7.67%. When diffusion capacity was evaluated, the DLCO percentage was calculated as a median of 68.50 (Q1–Q3: 51.00–80.50). The mean 6 min walk test distance was 391.18 ± 106.55 m. Thirteen patients (34.2%) were receiving pirfenidone therapy and 25 (65.8%) were receiving nintedanib therapy.

A total of 38 patients with a definitive diagnosis of idiopathic pulmonary fibrosis (IPF) according to the ATS/ERS 2022 criteria were included in the study. Eight patients were classified as anemic and 30 as non-anemic (Table 2).

In Table 2, when the anemic and non-anemic groups were compared, the ferritin level was 8.20 (3.60–238.50) μg/L in the anemic group and 39.80 (8.00–652.00) μg/L in the non-anemic group, and the difference was statistically significant (*p* = 0.037). The hemoglobin level was 11.93 ± 0.599 g/dL in the anemic group and 14.74 ± 1.04 g/dL in the non-anemic group (*p* < 0.001). The erythropoietin level was 22.68 ± 10.88 mIU/mL in the anemic group and 11.47 ± 6.36 mIU/mL in the non-anemic group (*p* = 0.035), and transferrin saturation was 10.25 ± 4.38% in the anemic group and 22.81 ± 10.28% in the non-anemic group (*p* < 0.001). In contrast, the hepcidin level did not differ between the groups (median: 30.47 (17.77–91.78) vs. 28.37 (10.56–60.21) ng/mL; *p* = 0.531). No significant difference was observed in other iron/hematological parameters (iron *p* = 0.184, UIBC *p* = 0.284, vitamin B12 *p* = 0.057, folate *p* = 0.480, serum transferrin *p* = 0.551).

**Figure 1:**
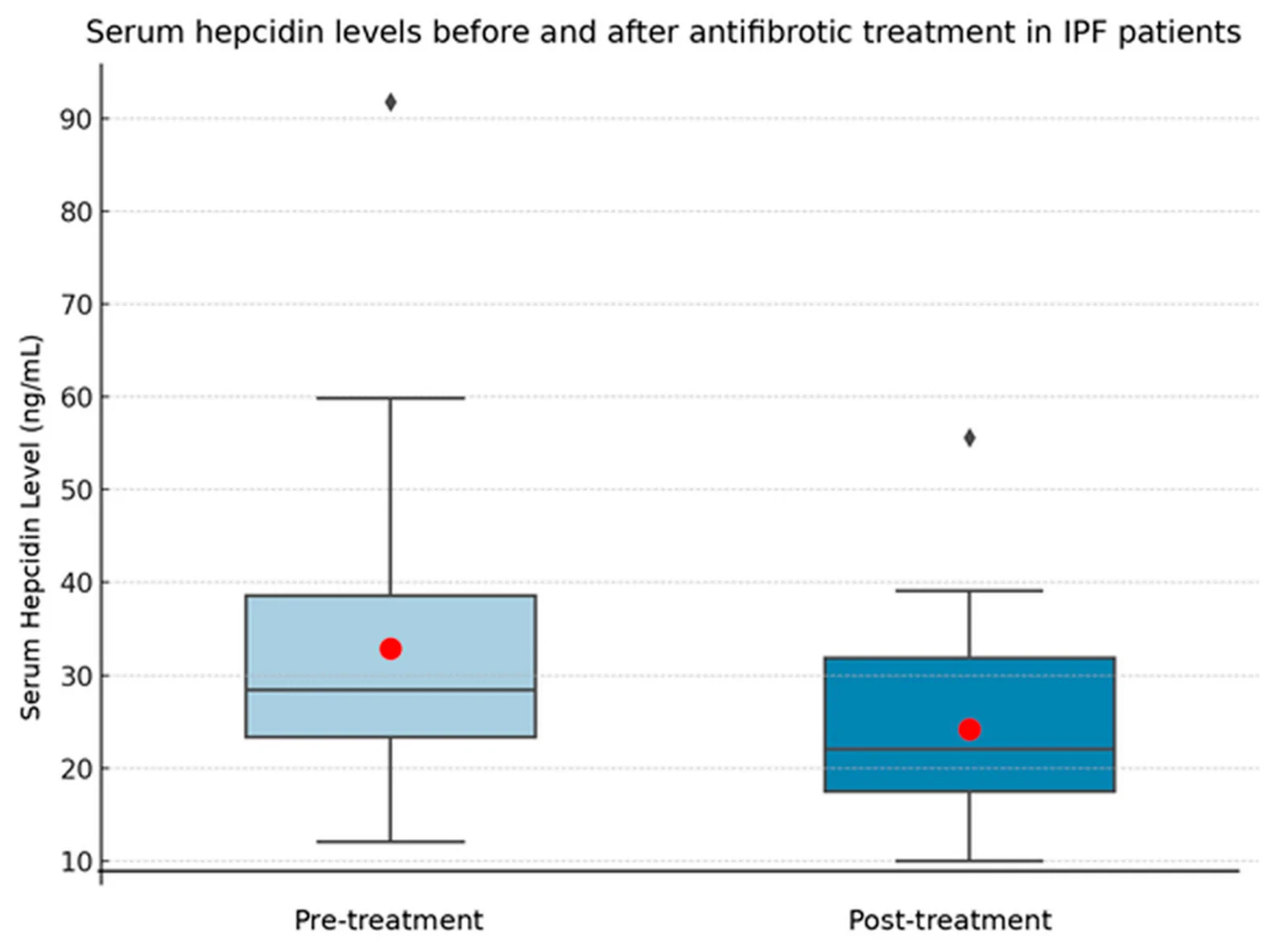
Box plot of serum hepcidin levels before and after antifibrotic treatment (red dots represent the means).

**Figure 2:**
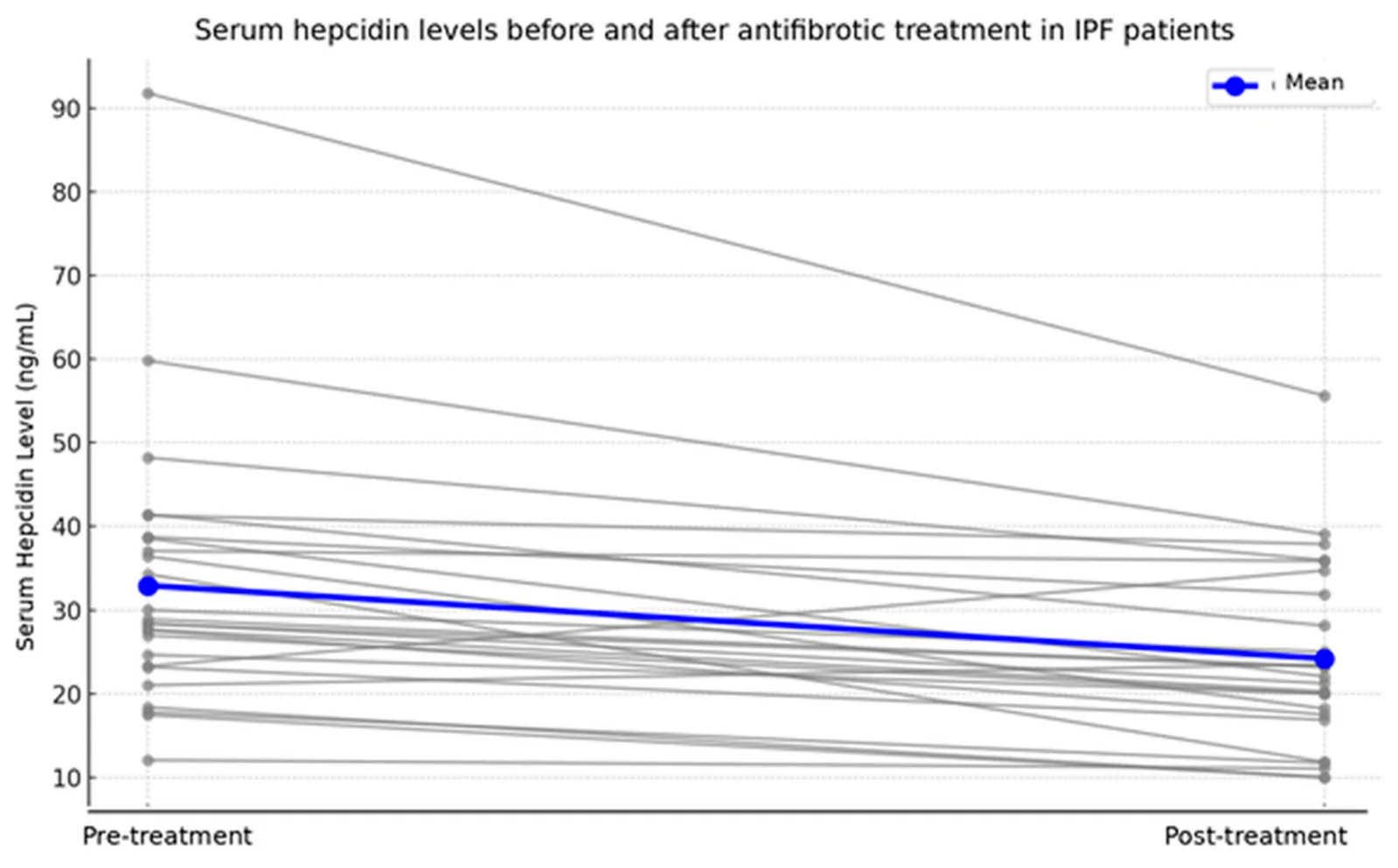
Individual and mean changes in serum hepcidin levels before and after antifibrotic treatment in IPF patients.

Serum hepcidin distributions and individual patient-level changes are presented in Figure 1 and Figure 2.

Before antifibrotic therapy, the mean ± SD serum hepcidin level was 33.00 ± 16.74 ng/mL, whereas it decreased to 24.41 ± 12.42 ng/mL at month 3 of therapy (paired-samples *t*-test, *p* < 0.001) (Table 3). In a post hoc sensitivity analysis excluding the eight participants with anemia at baseline, mean serum hepcidin decreased from 31.23 ± 13.72 ng/mL at baseline to 23.19 ± 11.93 ng/mL at month 3 among the remaining 30 participants (mean paired reduction, 8.04 ng/mL; 95% CI, 3.44–12.65; paired-samples *t*-test, t(29) = 3.57, *p* = 0.0013). A reduction in serum hepcidin levels was observed in 30 of 38 patients (78.9%) (Figure 2). Despite this change in hepcidin in Table 3, no statistically significant difference was detected in most of the other laboratory parameters examined in the comparisons between pre-treatment and month 3. For example, hemoglobin changed from 14.15 ± 1.51 to 14.28 ± 1.61 g/dL (*p* = 0.7024); CRP from 4.45 (3.00–6.00) to 3.50 (3.00–6.00) mg/L (*p* = 0.5874); and ESR from 18.16 ± 11.97 to 17.87 ± 14.38 mm/h (*p* = 0.9253). Similarly, PCT changed from 0.22 (0.19–0.26) to 0.24 (0.22–0.28) (*p* = 0.2380), HALP from 6.17 (3.79–8.41) to 5.53 (4.02–7.81) (*p* = 0.8382), SII from 543.79 (349.87–812.32) to 583.95 (453.94–869.40) (*p* = 0.9374), and PIV from 409.90 (281.44–587.69) to 452.44 (292.80–705.95) (*p* = 0.5717).

In the paired pre–post functional assessment, mean FVC increased from 73.83 ± 18.10% predicted at baseline to 77.74 ± 15.70% predicted at month 3 (mean paired increase, 3.90 percentage points; 95% CI, 2.03–5.77; paired-samples *t*-test, *p* < 0.001). DLCO increased from a median (Q1–Q3) of 68.50 (51.00–80.50) to 72.50 (60.00–83.75) % predicted (Wilcoxon signed-rank test, *p* < 0.001). In contrast, the 6 min walk distance changed from a median (Q1–Q3) of 370.00 (326.00–470.25) m to 400.00 (305.00–497.50) m and did not reach statistical significance (Wilcoxon signed-rank test, *p* = 0.0782).

In the Spearman correlation analysis performed in Table 4, no significant relationship was found between baseline hepcidin level and anemia-related parameters (all *p* > 0.05). For example, no statistically significant correlation was observed between hepcidin and hemoglobin (r = 0.020, *p* = 0.904), transferrin saturation (r = 0.018, *p* = 0.917), ferritin (r = −0.030, *p* = 0.857), and erythropoietin (r = 0.027, *p* = 0.873).

In Table 5, correlations between baseline hepcidin level and systemic inflammation indicators were examined. No statistically significant correlation was found between hepcidin and SII (r = 0.016, *p* = 0.924), CRP (r = 0.039, *p* = 0.818), erythrocyte sedimentation rate (r = −0.098, *p* = 0.558), and ferritin (r = −0.030, *p* = 0.857).

In Table 6, the change in hepcidin levels according to drug subgroups is presented. In the pirfenidone group (n = 13), hepcidin changed from 34.89 ± 12.26 to 27.02 ± 10.84, and this change was not statistically significant (*p* = 0.073). In the nintedanib group (n = 25), hepcidin decreased from 32.01 ± 18.82 to 23.05 ± 13.17, and the change was statistically significant (*p* < 0.001). However, no significant difference was found when the change in hepcidin (Δ) was compared between the pirfenidone and nintedanib groups (Welch *t*-test, *p* = 0.817).

Comparison of the pre- and post-treatment change (Δ) in laboratory parameters between groups in patients receiving pirfenidone and nintedanib treatment. Δ was calculated as “post-treatment − pre-treatment”. Continuous variables are presented as mean ± standard deviation or median (Q1–Q3) according to distribution characteristics. Between-group comparisons of Δ were performed using the Welch *t*-test when parametric assumptions were met and the Mann–Whitney U test when they were not met. For multiple comparisons, FDR (Benjamini–Hochberg) correction was applied and q-values were reported. No significant between-group differences in change were observed for PCT (*p* = 0.242), HALP (*p* = 0.397), SII (*p* = 0.939), or PIV (*p* = 0.939); all corresponding q-values were approximately 0.982 after FDR correction.

Spearman correlation analysis showed that greater reductions in serum hepcidin were associated with more favorable individual changes in FVC and DLCO, whereas no significant association was observed with the change in 6 min walk distance (Table 7).

**Figure 3:**
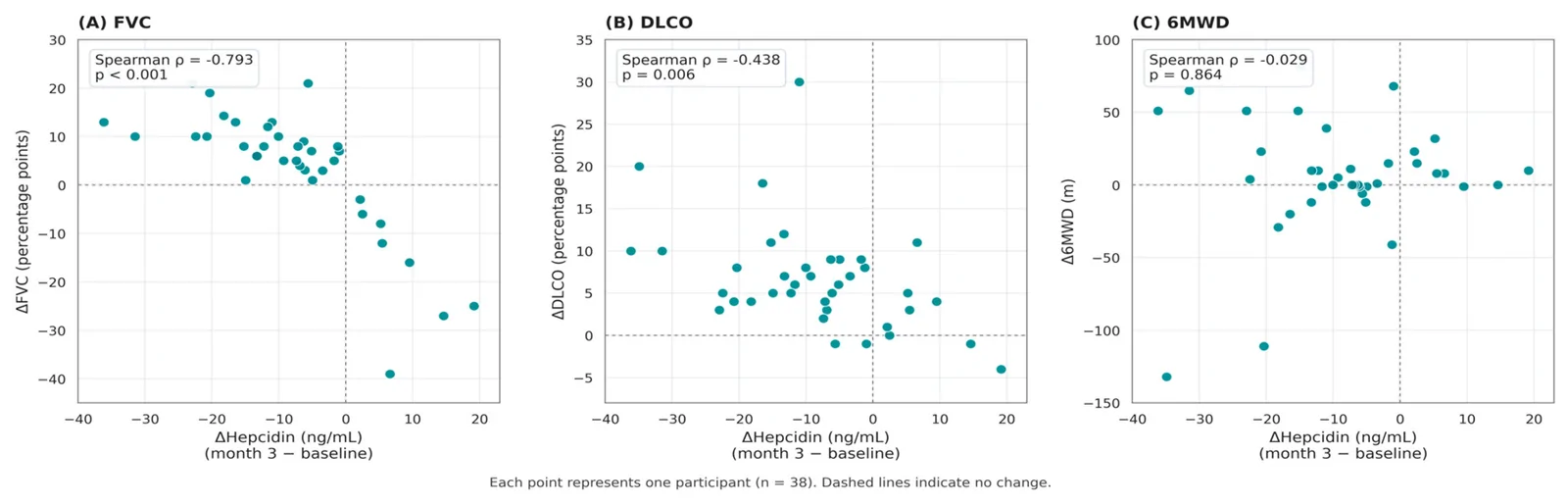
Associations between individual changes in serum hepcidin and functional outcomes over three months. Panels show Δhepcidin versus (**A**) ΔFVC, (**B**) ΔDLCO, and (**C**) Δ6 min walk distance. Each point represents one participant (n = 38); dashed lines indicate no change.

The relationships between three-month changes in serum hepcidin and changes in FVC, DLCO, and 6-min walk distance are illustrated in Figure 3.

Using change scores calculated as month 3 minus baseline, a multivariable linear regression model was constructed with Δhepcidin as the dependent variable and ΔFVC, ΔDLCO, Δ6 min walk distance, age, and biological sex entered simultaneously. The model was statistically significant (R^2^ = 0.546, adjusted R^2^ = 0.476; F(5, 32) = 7.71; *p* < 0.001). Only ΔFVC was independently associated with Δhepcidin (B = −0.848 ng/mL per percentage-point increase, 95% CI: −1.244 to −0.452; standardized β = −0.636; *p* < 0.001), whereas ΔDLCO (*p* = 0.262), Δ6 min walk distance (*p* = 0.831), age (*p* = 0.477), and biological sex (*p* = 0.610) were not statistically significant.

To further account for potential confounding by baseline disease severity, baseline FVC was added to the multivariable model as an additional covariate in a sensitivity analysis. The expanded model remained statistically significant (R^2^ = 0.594, adjusted R^2^ = 0.516; F(6, 31) = 7.57; *p* < 0.001). ΔFVC remained independently and inversely associated with Δhepcidin (B = −1.061 ng/mL per percentage-point increase, 95% CI: −1.505 to −0.617; standardized β = −0.796; *p* < 0.001), whereas baseline FVC did not reach statistical significance (B = −0.177, 95% CI: −0.366 to 0.012; *p* = 0.065). ΔDLCO (*p* = 0.574), Δ6 min walk distance (*p* = 0.806), age (*p* = 0.670), and biological sex (*p* = 0.760) were not independently associated with Δhepcidin. Thus, the association between ΔFVC and Δhepcidin was maintained after additional adjustment for baseline lung function.

As an additional sensitivity analysis, potential outliers in Δhepcidin and ΔFVC were identified using Tukey’s inner fences (Q1 − 1.5 × IQR and Q3 + 1.5 × IQR). After excluding four observations identified as potential outliers, the inverse association between Δhepcidin and ΔFVC remained statistically significant (n = 34, Spearman’s ρ = −0.713, *p* < 0.001). Furthermore, leave-one-out analyses yielded correlation coefficients ranging from −0.840 to −0.776, with all *p*-values < 0.001. These findings indicate that the observed association was not driven by the potential outliers or by any single participant.

## 4. Discussion

In this prospective real-life cohort, we found a statistically significant decrease in serum hepcidin levels in the third month of antifibrotic treatment (e.g., mean ± SD: from 33.00 ± 16.74 to 24.41 ± 12.42; *p* < 0.001). In parallel, FVC and DLCO increased significantly during the 3-month follow-up, whereas the change in 6 min walk distance was not statistically significant. The most pronounced decrease occurred in the patient with the highest baseline hepcidin level. In contrast, no significant change was observed in systemic inflammation/erythropoiesis indicators such as hemoglobin, CRP, and erythrocyte sedimentation rate. This pattern suggests that the hepcidin response may not be explained solely by the classical “acute-phase” or anemia-related axis.

Although statistically favorable short-term changes in FVC and DLCO were observed, these findings should be interpreted cautiously because of the short follow-up period, the absence of an untreated comparator group, and the potential influence of measurement variability and regression to the mean. Therefore, these changes cannot be attributed solely to antifibrotic therapy.

This finding is consistent with data from a previous study showing that hepcidin was elevated in patients with IPF compared with control groups and that this elevation was independent of anemia, EPO, and systemic inflammation parameters [16]. Taken together, the fact that elevated hepcidin accompanying IPF at the cross-sectional level can decrease longitudinally under antifibrotic treatment supports that hepcidin is not only a “state marker” but also has the potential to reflect a biological activity that can be modified by treatment.

Although hepcidin is primarily defined as a hepatic-derived systemic iron regulator, the demonstration of its expression in cells such as airway epithelium and alveolar macrophages in the lung suggests the presence of a regulatory layer specific to the pulmonary microenvironment [21,22]. In experimental models, disruption of the hepcidin/ferroportin axis has been found to be associated with thickening of interalveolar septa and marked structural changes in alveolar type II cells, indicating that this axis may be related to lung parenchymal integrity and remodeling processes [23]. In this framework, the increase in hepcidin in IPF and its decrease with treatment may be a biochemical reflection of the axis of alveolar epithelial injury–abnormal repair–fibroblast activation.

Classically, hepcidin is considered a component of an “acute-phase” response that can increase through the IL-6/STAT3 axis in inflammation [24,25,26]. However, the lack of significant change in systemic inflammation markers such as CRP/ESR in our study suggests that the decrease in hepcidin did not run in parallel with “reduction in systemic inflammation”, at least at the level we could measure. Similarly, the lack of significant change in hemoglobin makes it difficult to explain the decrease in hepcidin simply by an improvement in anemia.

Our main mechanistic interpretation is based on the hypothesis that hepcidin may be modulated through a regulatory network that intersects with “profibrotic pathways” in IPF. The critical role of the BMP/SMAD axis, particularly SMAD4, in hepatic hepcidin transcription has been shown experimentally; liver-specific loss of SMAD4 markedly reduces hepcidin expression [27]. On this biological basis, it has been reported that TGF-β1 can increase hepcidin mRNA expression in hepatocytes via a non-canonical mechanism mediated by ALK5/TβRII and involving phosphorylation of Smad1/5/8 [28]. In addition, activin B has been shown to be induced by inflammatory stimuli and to increase hepcidin through Smad1/5/8 signaling [29]. Notably, increased expression of activin B and follistatin in human IPF lung suggests that TGF-β superfamily signaling is active in the IPF microenvironment and may provide a theoretical bridge to hepcidin regulation [30].

In this context, experimental data showing that nintedanib can suppress profibrotic cellular processes (e.g., fibroblast proliferation/migration, etc.) by inhibiting PDGF/FGF/VEGF receptor tyrosine kinases [31] and that pirfenidone can reduce fibrotic targets such as collagen/HSP47 induced by TGF-β1 [32,33] support the hypothesis that the decrease in hepcidin under antifibrotic treatment may be a pharmacodynamic response consistent with a “reduction in fibrotic signaling burden”. This hypothesis is particularly valuable in explaining hepcidin changes independent of systemic inflammation or anemia, because mechanistic evidence indicates that hepcidin regulation can be shaped not only via IL-6/STAT3 but also through the TGF-β superfamily/BMP–SMAD intersection [27,29].

The absence of a statistically significant change in PCT, HALP, SII, and PIV values after treatment in our study may be considered consistent with the fact that these indices often behave as a “snapshot” of basal systemic inflammation/immune status rather than as dynamic indicators of treatment response in clinical practice. Indeed, although associations between peripheral blood cell counts and composite inflammation indices with prognosis and functional status in IPF have been shown, it has been reported that annual changes in these parameters may not always show a significant correlation with clinical/functional variables [34].

Similarly, studies reporting that SII can be used to predict mortality in IPF have focused mainly on baseline SII level, and the lack of significant change after short-term treatment does not exclude the value of these indices in terms of “prognostic classification”; however, it suggests that their sensitivity in follow-up/response assessment may be limited [13]. In contrast, the significant change in hepcidin level after treatment in our study indicates that hepcidin may be closer to the target (closer to the mechanism) and potentially a more sensitive follow-up biomarker candidate compared with these hemogram-derived indices. Hepcidin is the main regulator of iron homeostasis and is also closely related to inflammation; it has been shown that hepcidin levels increase with inflammatory stimuli and that especially IL-6-mediated signaling induces hepcidin expression [24,26,35].

In the context of IPF, it has been reported that hepcidin may be higher in patients with IPF than in the control group and that this increase may be independent of anemia and classical systemic inflammation parameters; this finding suggests that hepcidin may reflect a biological axis (iron metabolism–inflammation interaction) independent of hemogram-based inflammation indices [16]. In addition, it has been reported that hepcidin can behave like a “treatment-sensitive” acute-phase marker in different clinical contexts (e.g., it can change together with treatment in bacteremia) [36]. In terms of lung pathophysiology, experimental data are also available showing that disruption of the hepcidin/ferroportin axis can be associated with pulmonary iron loading and restrictive lung disease [37]. Within this literature, when our significant change in hepcidin in the pre–post analysis is evaluated together with the lack of change in PCT, HALP, SII, and PIV, it supports that hepcidin may be a more sensitive and clinically more valuable candidate biomarker for capturing treatment effects.

In the drug-based subanalysis (pirfenidone n = 13, nintedanib n = 25), no significant difference was detected in any parameter in the comparisons of Δ values between groups. For example, the change in hepcidin was −7.88 ± 14.48 in the pirfenidone group and −8.96 ± 11.51 in the nintedanib group, and the between-group difference was not significant (Welch *t*-test, *p* = 0.817; q = 0.982 after FDR). Similarly, no between-group Δ difference was observed in inflammation indices (e.g., PCT *p* = 0.242, HALP *p* = 0.397, SII *p* = 0.939, PIV *p* = 0.939; all q-values ≈ 0.982 after FDR). Therefore, our findings suggest that the change in hepcidin may have the potential to reflect the general follow-up dynamics in patients with IPF receiving antifibrotic therapy, rather than being an effect that “distinguishes drug selection”. From a clinical perspective, the short-term decrease in hepcidin under antifibrotic therapy suggests that hepcidin may be a potential “pharmacodynamic biomarker”.

The main limitations of our study are its single-center design, limited sample size, and relatively short follow-up period. Because of the small number of patients in the pirfenidone subgroup (n = 13), the between-group comparison had limited statistical power. Therefore, the absence of a statistically significant difference should not be interpreted as evidence of equivalence between pirfenidone and nintedanib, and these subgroup findings should be considered exploratory. Although pulmonary function testing was performed in the same laboratory using the same equipment and standardized procedures at both visits, the absence of repeated pre-treatment measurements and an untreated comparator group means that measurement variability, test familiarization, and regression to the mean cannot be excluded. Accordingly, the short-term increases in FVC and DLCO should be regarded as exploratory and do not, on their own, demonstrate fibrosis reversal or a direct treatment effect. In addition, the fact that IL-6, the hepcidin/ferroportin axis at the lung tissue level, and profibrotic mediators (TGF-β1, activin B, BMP6, etc.) were not measured directly limits linking the findings to pathophysiological mechanisms. In this framework, it is thought that future multicenter prospective studies including larger patient populations and longer follow-up periods will more clearly demonstrate the prognostic value of hepcidin in monitoring response to antifibrotic therapy.

In the absence of an untreated comparator group, the observed decrease in serum hepcidin cannot be attributed exclusively to antifibrotic therapy; natural temporal variation and the effects of unmeasured confounding factors cannot be excluded.

Although nutritional status and gastrointestinal adverse effects were clinically monitored, dietary iron intake was not quantitatively assessed using a standardized dietary instrument; therefore, the possible influence of subtle changes in dietary iron intake on serum hepcidin levels cannot be completely excluded.

In conclusion, the significant short-term decrease in serum hepcidin levels in patients with IPF receiving antifibrotic therapy indicates that hepcidin may be a biomarker candidate that can be modified by treatment in IPF and is relatively independent of the inflammation/anemia axis, and that it may be used as an objective indicator of biological response to antifibrotic therapy. This finding indicates that the clinical and translational importance of hepcidin for monitoring treatment response in IPF management should be clarified with further and broader studies. Serum hepcidin decreased during the first three months following the initiation of antifibrotic therapy, and greater reductions were associated with more favorable short-term changes in FVC and DLCO, but not in 6 min walk distance. These exploratory findings support further investigation of serum hepcidin as a candidate treatment-response biomarker but do not establish its clinical utility.

## Figures and Tables

**Table 1:** Baseline demographic and clinical characteristics of IPF patients (n = 38).

Variable	Overall (n = 38)
Age, years	71.47 ± 6.94
Sex (male/female), n (%)	20/18 (52.6%/47.4%)
Anemia (WHO criteria: female < 12, male < 13 g/dL), n (%)	8 (21.1%)
Smoking Pack-years, median (Q1–Q3)	15.00 (0.00–46.25)
Charlson Comorbidity Index, median (Q1–Q3)	3.00 (3.00–4.00)
FVC, % predicted	73.83 ± 18.10
FEV1, % predicted	82.22 ± 19.68
FEV1/FVC, %	85.61 ± 7.67
DLCO, % predicted, median (Q1–Q3)	68.50 (51.00–80.50)
6 min walk distance, m	391.18 ± 106.55
Pirfenidone, n (%)	13 (34.2%)
Nintedanib, n (%)	25 (65.8%)

Data are presented as mean ± SD or median (Q1–Q3), as appropriate. Categorical variables are shown as n (%).

**Table 2:** Comparison of hepcidin and anemia-related parameters between anemic and non-anemic IPF patients.

Variable	Anemic (WHO Criteria: Female < 12, Male < 13 g/dL) (n = 8)	Non-Anemic (n = 30)	*p*
Age (years) Mean ± SD	75.00 ± 5.01	70.53 ± 7.15	0.060
Hepcidin (ng/mL) Median (min–max)	30.47 (17.77–91.78)	28.37 (10.56–60.21)	0.531
Smoking (pack-years)	30.00 (0.00–60.00)	15.00 (0.00–50.00)	0.864
CCI *	4.00 (3.00–5.00)	3.00 (2.00–5.00)	0.511
Ferritin (μg/L) Median (min–max)	8.20 (3.60–238.5)	39.80 (8.00–652.0)	0.037
Hemoglobin (g/dL)	11.93 ± 0.599	14.74 ± 1.04	<0.001
Iron (Fe) (μg/dL) Mean ± SD	50.57 ± 36.78	72.69 ± 33.23	0.184
UIBC (μg/dL) Mean ± SD	316.0 ± 101.9	269.2 ± 64.04	0.284
Erythropoietin (mIU/mL) Mean ± SD	22.68 ± 10.88	11.47 ± 6.36	0.035
Vitamin B12 (ng/L) Median (min–max)	463.0 (275.0–1500)	214.0 (66.00–942.0)	0.057
Folate (μg/L) Median (min–max)	6.40 (3.60–15.70)	7.50 (3.00–21.20)	0.480
Serum transferrin (g/L) Median (min–max)	2.40 (2.00–2.74)	2.54 (1.66–4.11)	0.551
Transferrin saturation (%) Mean ± SD	10.25 ± 4.38	22.81 ± 10.28	<0.001

*: CCI: Charlson Comorbidity Index.

**Table 3:** Pre- and post-treatment comparison of hepcidin, functional parameters, and analyzed laboratory biomarkers in IPF patients.

Parameter	Pre-Treatment (Baseline)	Post-Treatment (Month 3)	Test	*p*
Hepcidin (ng/mL)	33.00 ± 16.74	24.41 ± 12.42	Paired *t*-test	<0.001
FVC (% predicted)	73.83 ± 18.10	77.74 ± 15.70	Paired *t*-test	<0.001
DLCO (% predicted)	68.50 (51.00 to 80.50)	72.50 (60.00 to 83.75)	Wilcoxon signed-rank	<0.001
6 min walk distance (m)	370.00 (326.00 to 470.25)	400.00 (305.00 to 497.50)	Wilcoxon signed-rank	0.0782
WBC (×10^9^/L)	8.92 ± 1.75	9.50 ± 2.55	Paired *t*-test	0.2192
Monocytes (%)	8.59 ± 1.49	7.95 ± 2.52	Paired *t*-test	0.2269
Neutrophils (%)	59.75 ± 9.38	62.37 ± 9.96	Paired *t*-test	0.2346
PCT (%)	0.22 (0.19 to 0.26)	0.24 (0.22 to 0.28)	Wilcoxon signed-rank	0.2380
Neutrophils (NE, ×10^9^/L)	5.20 (4.22 to 6.22)	5.62 (4.58 to 6.68)	Wilcoxon signed-rank	0.2454
Lymphocytes (%)	27.49 ± 8.45	25.43 ± 8.95	Paired *t*-test	0.3294
Eosinophils (%)	3.38 ± 2.46	3.03 ± 1.95	Paired *t*-test	0.4443
Basophils (%)	0.65 ± 0.32	0.62 ± 0.30	Paired *t*-test	0.6664
Basophils (BA, ×10^9^/L)	0.10 (0.00 to 0.10)	0.10 (0.00 to 0.10)	Wilcoxon signed-rank	0.6698
Monocytes (MO, ×10^9^/L)	0.78 (0.60 to 0.90)	0.69 (0.55 to 1.05)	Wilcoxon signed-rank	0.6782
Hemoglobin (g/dL)	14.15 ± 1.51	14.28 ± 1.61	Paired *t*-test	0.7024
Eosinophils (EO, ×10^9^/L)	0.28 (0.20 to 0.49)	0.28 (0.10 to 0.42)	Wilcoxon signed-rank	0.7063
Platelet (PLT, ×10^9^/L)	248.82 ± 48.37	253.85 ± 67.03	Paired *t*-test	0.7173
Hematocrit (HCT, %)	42.35 (39.55 to 44.00)	42.20 (38.62 to 46.27)	Wilcoxon signed-rank	0.7964
RDW (%)	14.70 (13.72 to 15.72)	14.40 (13.70 to 15.55)	Wilcoxon signed-rank	0.8034
Lymphocytes (LY, ×10^9^/L)	2.41 ± 0.79	2.46 ± 1.05	Paired *t*-test	0.8326
MCHC (g/dL)	33.55 (32.60 to 34.55)	33.45 (32.62 to 34.10)	Wilcoxon signed-rank	1.0000
CRP (mg/L)	4.45 (3.00 to 6.00)	3.50 (3.00 to 6.00)	Wilcoxon signed-rank	0.5874
RBC (×10^12^/L)	4.70 (4.54 to 5.10)	4.79 (4.44 to 5.20)	Wilcoxon signed-rank	0.8522
ESR (mm/h)	18.16 ± 11.97	17.87 ± 14.38	Paired *t*-test	0.9253
HALP	6.17 (3.79 to 8.41)	5.53 (4.02 to 7.81)	Wilcoxon signed-rank	0.8382
SII	543.79 (349.87 to 812.32)	583.95 (453.94 to 869.40)	Wilcoxon signed-rank	0.9374
PIV	409.90 (281.44 to 587.69)	452.44 (292.80 to 705.95)	Wilcoxon signed-rank	0.5717

Data are presented as mean ± SD for parameters analyzed with a paired *t*-test and as a median (Q1–Q3) for parameters analyzed with the Wilcoxon signed-rank test; *p*-values are two-sided. Abbreviations: FVC, forced vital capacity; DLCO, diffusing capacity of the lung for carbon monoxide; WBC, white blood cell count; PCT, plateletcrit; NE, neutrophils; BA, basophils; MO, monocytes; EO, eosinophils; PLT, platelet count; HCT, hematocrit; RDW, red cell distribution width; LY, lymphocytes; MCHC, mean corpuscular hemoglobin concentration; CRP, C-reactive protein; RBC, red blood cell count; ESR, erythrocyte sedimentation rate; HALP, hemoglobin–albumin–lymphocyte–platelet score; SII, systemic immune-inflammation index; PIV, pan-immune-inflammation value.

**Table 4:** Correlation between baseline hepcidin levels and anemia parameters in IPF patients (Spearman’s rho).

Variable	r	*p*
UIBC (μg/dL)	−0.029	0.862
Vitamin B12 (ng/L)	0.049	0.769
Iron (μg/dL)	0.112	0.504
Ferritin (μg/L)	−0.030	0.857
Folate (μg/L)	−0.195	0.241
Hemoglobin (g/dL)	0.020	0.904
Transferrin saturation (%)	0.018	0.917
Serum transferrin (g/L)	−0.167	0.315
Erythropoietin (mIU/mL)	0.027	0.873

**Table 5:** Correlation between baseline hepcidin levels and systemic inflammation parameters in IPF patients (Spearman’s rho).

Variable	r	*p*
SII *	0.016	0.924
Ferritin (μg/L)	−0.030	0.857
C-reactive protein (mg/L)	0.039	0.818
Erythrocyte sedimentation rate (mm/h)	−0.098	0.558

*: SII, systemic immune-inflammation index.

**Table 6:** Change in hepcidin values in patients receiving pirfenidone and nintedanib treatment.

Group	n	Pre (Mean ± SD)	Post (Mean ± SD)	Δ (Mean ± SD)	Within-Group *p*
Pirfenidone	13	34.89 ± 12.26	27.02 ± 10.84	−7.88 ± 14.48	0.073
Nintedanib	25	32.01 ± 18.82	23.05 ± 13.17	−8.96 ± 11.51	<0.001

Comparison of the pre- and post-treatment change in hepcidin (Δ) between groups in patients receiving pirfenidone and nintedanib treatment: *p* = 0.817 with the Welch *t*-test.

**Table 7:** Correlations between individual changes in serum hepcidin and functional outcomes over three months (Spearman’s rho).

Correlation Pair	n	Spearman’s ρ	*p*-Value
ΔHepcidin–ΔFVC	38	−0.793	<0.001
ΔHepcidin–ΔDLCO	38	−0.438	0.006
ΔHepcidin–Δ6 min walk distance	38	−0.029	0.864

## Data Availability

The data presented in this study are available from the corresponding author upon reasonable request. The data are not publicly available due to privacy and ethical restrictions concerning participant confidentiality.
